# A Social Media Infodemic-Based Prediction Model for the Number of Severe and Critical COVID-19 Patients in the Lockdown Area

**DOI:** 10.3390/ijerph19138109

**Published:** 2022-07-01

**Authors:** Qi Yan, Siqing Shan, Menghan Sun, Feng Zhao, Yangzi Yang, Yinong Li

**Affiliations:** 1 School of Economics and Management, Beihang University, Beijing 100191, China; shansiqing@buaa.edu.cn (S.S.); smh_gabriella@buaa.edu.cn (M.S.); zhao_feng@buaa.edu.cn (F.Z.); ymyang@buaa.edu.cn (Y.Y.); liyinong@buaa.edu.cn (Y.L.); 2 Beijing Key Laboratory of Emergency Support Simulation Technologies for City Operation, Beijing 100191, China

**Keywords:** COVID-19, prediction model, machine learning, sentiment polarity, social media

## Abstract

Accurately predicting the number of severe and critical COVID-19 patients is critical for the treatment and control of the epidemic. Social media data have gained great popularity and widespread application in various research domains. The viral-related infodemic outbreaks have occurred alongside the COVID-19 outbreak. This paper aims to discover trustworthy sources of social media data to improve the prediction performance of severe and critical COVID-19 patients. The innovation of this paper lies in three aspects. First, it builds an improved prediction model based on machine learning. This model helps predict the number of severe and critical COVID-19 patients on a specific urban or regional scale. The effectiveness of the prediction model, shown as accuracy and satisfactory robustness, is verified by a case study of the lockdown in Hubei Province. Second, it finds the transition path of the impact of social media data for predicting the number of severe and critical COVID-19 patients. Third, this paper provides a promising and powerful model for COVID-19 prevention and control. The prediction model can help medical organizations to realize a prediction of COVID-19 severe and critical patients in multi-stage with lead time in specific areas. This model can guide the Centers for Disease Control and Prevention and other clinic institutions to expand the monitoring channels and research methods concerning COVID-19 by using web-based social media data. The model can also facilitate optimal scheduling of medical resources as well as prevention and control policy formulation.

## 1. Introduction

Considering the COVID-19 treatment practices in countries such as China, Italy, Spain, the United Kingdom, and the United States, severe and critical patients with COVID-19 in lockdown areas have relatively high mortality rates. Improvements in the recovery rate of the numbers of severe and critical COVID-19 patients is a critically important factor for effective control of the epidemic. The effective treatment of severe and critical COVID-19 patients requires massive medical resources, including setting up intensive care units (ICU) wards, equipping professional equipment and facilities (e.g., ventilators, EMCO), and deploying technical medical personnel. The increasing numbers of severe and critical COVID-19 patients are of great uncertainty, significantly disrupting the normal clinical treatment order of hospitals. If the allocation of medical resources is not timely and adequate, the regular and orderly treatment of patients will be seriously interrupted. Accurate prediction of the number of severe and critical COVID-19 patients helps to solve problems such as disordered treatment, unreasonable deployment of medical personnel, and inability to arrange professional medical equipment and facilities in advance. Therefore, patient-oriented rescue measures can improve the effectiveness of treatment and help policymakers formulate tailored policy measures for epidemic prevention and control. Accurate prediction of the number of severe and critical COVID-19 patients is an urgent issue to combat the epidemic of COVID-19.

The purpose of this study is to predict the number of COVID-19 severe and critical patients in a lockdown area (Hubei Province, China) via data from the social media platform, Sina Weibo. Social media, also named social sensor, has the advantages of timeliness, openness, and easy access. Nguyen et al. (2020) studied the influence of customer emotions transited in social media on the investment strategies of investors and enterprise value [1]. He et al. (2018) investigated the way non-local enterprises utilized social media to promote effective purchases and to improve customer relationships after a crisis in public relations [2]. Severe and critical COVID-19 patients in the lockdown area are also popular topics on social media. People post their needs and concerns on social media, so that the “infodemic” quickly generates and spreads. Such social media data can reflect valuable information on severe and critical COVID-19 patients in a timely and effective manner, including the number of patients and the emotional intensity of patients. Recognizing, understanding, and controlling the “infodemic” helps to predict the development of the epidemic. This study uses social media data to build a prediction model with a forecast horizon, to predict the number of severe and critical COVID-19 patients in the lockdown area. The prediction model is capable of solving the predict uncertainty issues, and the forecast horizon of the model can save time for medical institutions, medical staff, and policymakers in advance to properly predict the disease epidemic and formulate prevention measurements.

This study contributes to the existing literature in three aspects. First, in terms of the research perspective, this study uses social media data to predict the number of severe and critical COVID-19 patients in the lockdown area, which is also a popular topic on social media. Second, in terms of research data, this study uses both real-time social media data and published consensus data on severe and critical patients for making predictions. Social media data are advantageous as they involve large numbers of people’s sensors, provide timely information, and facilitate intense public discussion and public attention. Third, in terms of the research method, it proposed an improved machine learning prediction model based on the Hidden Markov model (HMM). This model can accurately predict the number of severe and critical COVID-19 patients in the lockdown area. Currently, more studies are focusing on exploring and predicting the severity of the COVID-19 epidemic. For example, Menni et al. (2020) used a symptom tracker to predict the number of potentially infected people by establishing a logistic regression model. COVID-19 symptom data in their study was collected through a smartphone app [3]. Yan et al. (2020) proposed a logically straightforward and easy decision rule to predict patients at the highest risk. Data were sourced from a blood sample database of COVID-19 infected patients in Wuhan, China [4]. Based on an exponential growth model, Tsang et al. (2020) estimated the impact of different definitions of COVID-19 confirmed cases on the epidemic’s curve and transmission parameters and further predicted the number of new infections [5]. Based on a stochastic propagation dynamics model, Kucharski et al. (2020) predicted the dynamics of the outbreak and the future downward trend of the COVID-19 epidemic [6]. Yang et al. (2020) constructed an improved Susceptible-Exposed-Infectious-Removed (SEIR) model and Long-Short-Term-Memory (LSTM) neural network to predict the development trend of the COVID-19 epidemic under public health policy interventions [7]. The study used data from confirmed diagnoses published daily in China and travel data for public transportation. Based on consensus data of confirmed cases, Leung et al. (2020) proposed the Susceptible–Infectious–Recovered model to study the impact assessment of the dissemination and severity of COVID-19 under public health interventions outside Hubei, China [8]. Based on Wuhan’s real-time mobility data and detailed consensus data of COVID-19 infection cases, Chinazzi et al. (2020) explored the role of imported COVID-19 infection cases in the spread of Chinese cities and measured the impact of lockdown measures on the COVID-19 epidemic; their study results showed that the interventions implemented in China achieved plausible progress in slowing down the spread of COVID-19 locally [9]. Tian et al. (2020) also investigated the spread and control of the COVID-19 epidemic with data including case reports, human activities, and public health interventions; they found that the national emergency response appeared to combat the growth of the COVID-19 epidemic in China, to a large extent, and limit the scale of the epidemic, avoiding hundreds of thousands of potential cases by February 19 (day 50) [10]. In addition, Jia et al. (2020) measured the risk of regional outbreaks from the perspective of population mobility using mobile phone data [11]. By collecting clinical data from two New York hospitals, Cummings et al. (2020) studied epidemiological characteristics and evolution of severe and critical patients [12]. Álvarez-Mon et al. (2021) created an individualized analysis model of the risk of ICU admission or death for COVID-19 patients as a tool for the rapid clinical management of hospitalized patients in order to achieve resilience of medical resources [13]. To predict the time of the outbreak peak and the ICU beds required, Moghadas et al. (2020) simulated a COVID-19 outbreak by parameterizing U.S. demographics [14]. The results showed that the ICU capacity was insufficient in responding to this rapid outbreak. Policies that encourage self-isolation, may delay the peak of the epidemic and provide a time window for emergency mobilization to expand hospital capacity. Mohsin et al. (2021) identified several vital lifestyles and comorbidity-related risk factors of severe-critical COVID-19 [15]. HMM has gained growing popularity in the fields of bioinformatics, fault diagnosis, and disease prediction. Based on HMM, Perveen et al. (2019) used primary electronic health care cases to predict the risk of developing diabetes within 8 years and identify various factors affecting the risk of disease [16]. Barra et al. (2020) proposed a method for quantifying the number and duration of migraine attacks based on the patient’s diary [17]. This model enables researchers to procure data with high inter-study validity. In addition, social media data have become one of the important data sources for scientific research [18], and the significant amount of hidden but valuable information it contains is worth mining and analyzing. Deng et al. (2018) comprehensively investigated the relationship between microblog sentiment and stock returns at the market and individual stock levels [19]. Roy et al. (2020) constructed an algorithm model called the “Suicide Artificial Intelligence Prediction Heuristic”, to predict the future risk of suicidal thoughts by analyzing public Twitter data [20]. Gan et al. (2022) studied differences in posting patterns and user behaviors between men and women during the COVID-19 epidemic through social media [21].

## 2. Materials and Methods

### 2.1. Data Description

**Figure 1:**
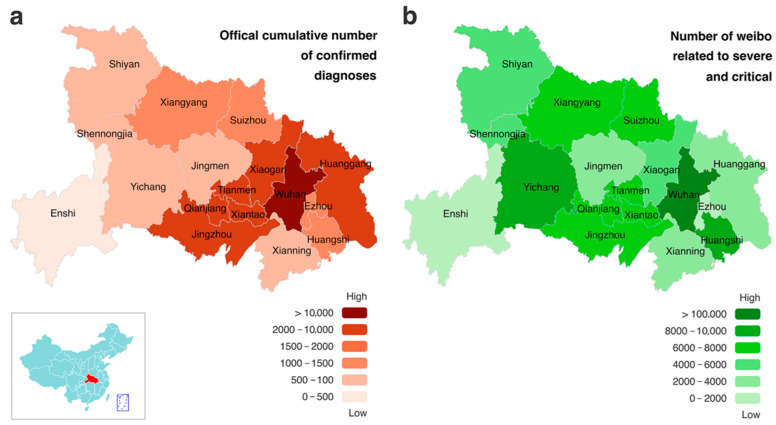
Geographic distribution of the number of perceived severe and critical COVID-19 patients and the number of confirmed cases by region in Hubei Province. Note: (**a**) Number of confirmed COVID-19 patients in Hubei Province during the lockdown period reported by consensus statistics. (**b**) Number of severe and critical patients related social media texts (abbreviated as SC-related social media texts) in Hubei Province during the lockdown period. The darker colors in the map indicate a larger number of COVID-19 patients in the region, indicating a more severe epidemic and a higher number of confirmed patients. In both maps, Wuhan is the darkest region, indicating that the epidemic situation shown by the number of SC-related social media texts is highly consistent with the actual situation. In Shiyan, Xiangyang, Suizhou, Jingmen, Enshi, Xianning, Huangshi, and other cities, there is a correlation between the number of SC-related social media texts and the cumulative number of confirmed COVID-19 patients, which shows a similar color change trend in the map; this indicates that the geographical distribution of the number of SC-related social media texts can effectively reflect the severity of COVID-19 in different regions. The number of SC-related social media texts in Xiaogan, Huanggang, Jingzhou, and other regions reflects the degree of the epidemic situation to be lower than the actual diagnosis, and higher than the actual diagnosis in Yichang. The trend difference in some cities may be due to inconsistencies in the number of COVID-19 patients and severe and critical COVID-19 patients. Since the information on geographic location collected from social media only contains 13 prefecture-level cities, data for the three county-level cities of Xiantao, Qianjiang, and Tianmen are considered as one area when merged with Jingzhou, and data for the Shennongjia forest area are considered as one area when merged with Shiyan.

This paper aims to predict the number of perceived severe and critical COVID-19 patients from web-based social media data combined with officially published data and improve the predictions of severe and critical patient numbers in the lockdown area. The lockdown in Hubei Province began on 23 January 2020, and ended on 24 March 2020. Hubei Province began publishing consensus data on severe and critical patients on 20 January 2020, which are taken from public datasets that are freely available online. Therefore, the period for data collection in this study was from 0:00 on 20 January 2020 to 24:00 on 24 March 2020. To measure the prevalence of COVID-19 on social media, we collected and analyzed social media data through Sina Weibo (one of the largest web-based social media platforms in China) API (Application Programming Interface). We purchased the data collection service from Gooseeker. Gooseeker is an authorized API of Sina Weibo. Specifically, this study collected social media data that contains at least one of the following keywords including “FEVER”, “GETTING HEAT”, “INFECT”, “PNEUMONIA”, “COUGH”, “VIRUS”, and “ISOLATION”. The post should be released from 20 January 2020 to 24 March 2020, by a blogger that is registered in Hubei Province. The whole data set amounts at 1,076,174 items, and each social media text contains information such as content, blogger name, posting time, and URL. The geographical distribution of social media data is shown in Figure 1. Social media data contains COVID-19-related microblogs published by two types of patients: bloggers who already knew about self-infection and others who experienced similar COVID-19 symptoms but were unsure about infection. As a social sensor, social media data can reflect the characteristics and emotions of COVID-19 patients or potential patients in reality.

### 2.2. Research Model

Based on HMM, this study builds a supervised machine learning model by considering the consensus data of severe and critical COVID-19 patients, the number of perceived severe and critical patients from social media, and the sentiment polarity of COVID-19-related social media texts; this model can realize the phased and horizontal prediction of severe and critical patient numbers in the lockdown area. The innovation of this study lies in three aspects. First, we construct an improved prediction model based on HMM. Many studies [22,23,24,25] have shown that machine learning works better in analyzing and processing nonlinear data. Compared with the time series model and regression model, HMM is a data-driven algorithm and has a substantial capacity to approximate nonlinear functions which can be used to satisfactorily predict nonlinear, time-varying data; moreover, it is not necessary to choose the form of function artificially in HMM, which helps to explore the rules that are difficult to be found in the traditional linear regression model. Therefore, HMM can better adapt to the characteristics of complex data structure, which is consistent with our data feature in this paper; this machine learning model can realize the phased and prospective prediction of COVID-19 severe and critical patients in the lockdown area. The forecast horizon in the model guarantees more time for clinical treatment preparation. The model helps to make multiple predictions at various time points during the lockdown period. Second, by mining COVID-19-related information in social media data, we identified two key prediction indicators driven by social media data: the number of perceived severe and critical COVID-19 patients and the sentiment polarity of COVID-19-related social media texts. Compared with the studies that used official published data, it was verified that these two indicators improve the accuracy of the prediction of quantity. Third, we propose a COVID-19 prevention and control method, which can help the medical system to formulate treatment plans by predicting the number of severe and critical patients, dispatching medical personnel, and formulating prevention and control policies to enhance the quality of work; it can assist medical staff to perform early detection and intervention on patients, effectively reducing the case fatality rates as a result. Thus, this study provides a theoretical and methodical framework to utilize social media data in the prevention and control of the COVID-19 epidemic.

**Figure 2:**
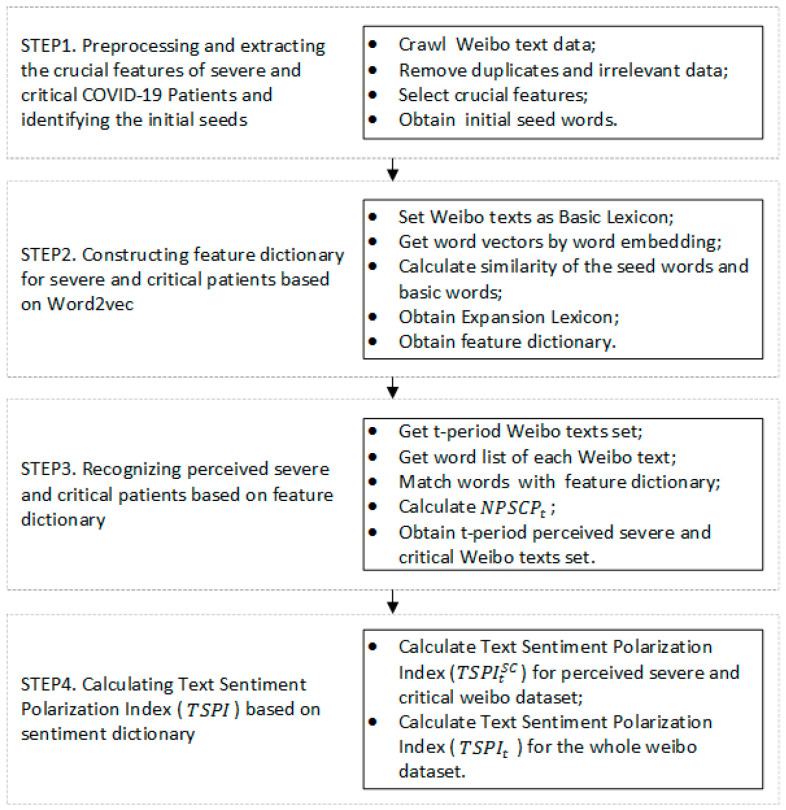
The conceptual framework of the integrated model for $NPSCP_{t}$, $TSPI_{t}$ and $TSPI_{t}^{SC}$.

**Figure 3:**
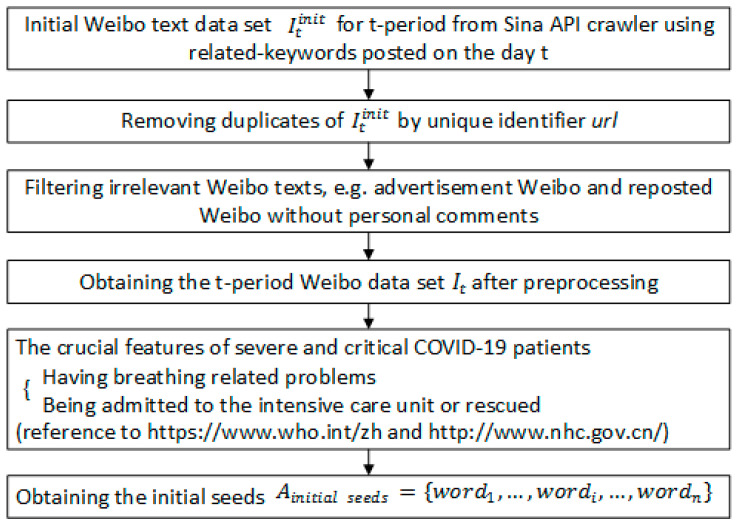
The first step of the integrated model.

**Figure 4:**
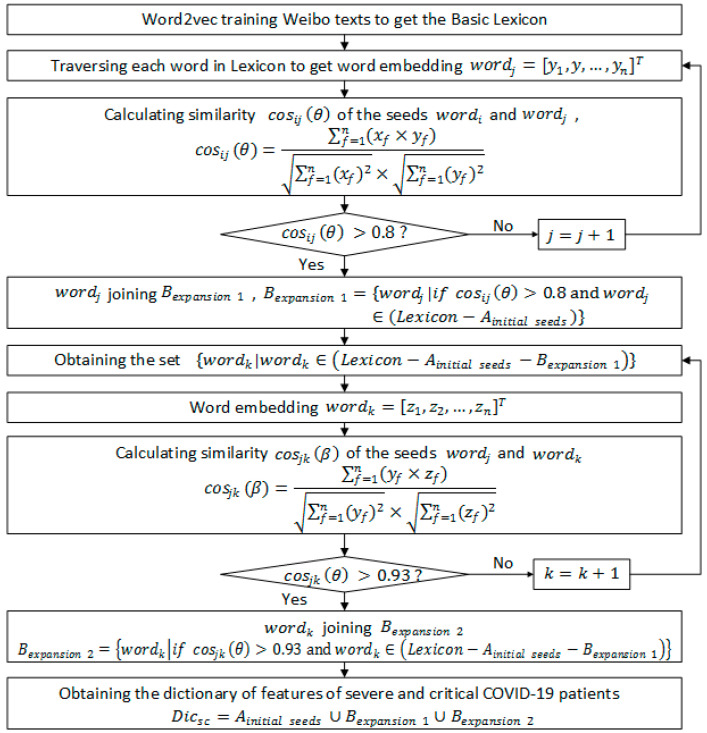
The second step of the integrated model.

**Figure 5:**
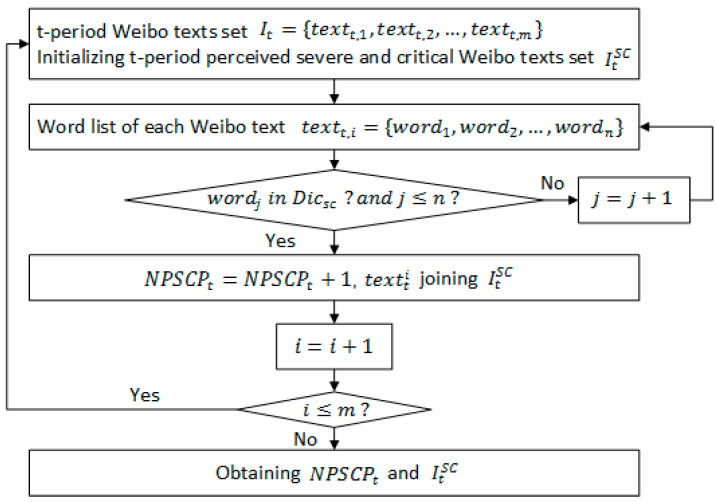
The third step of the integrated model.

**Figure 6:**
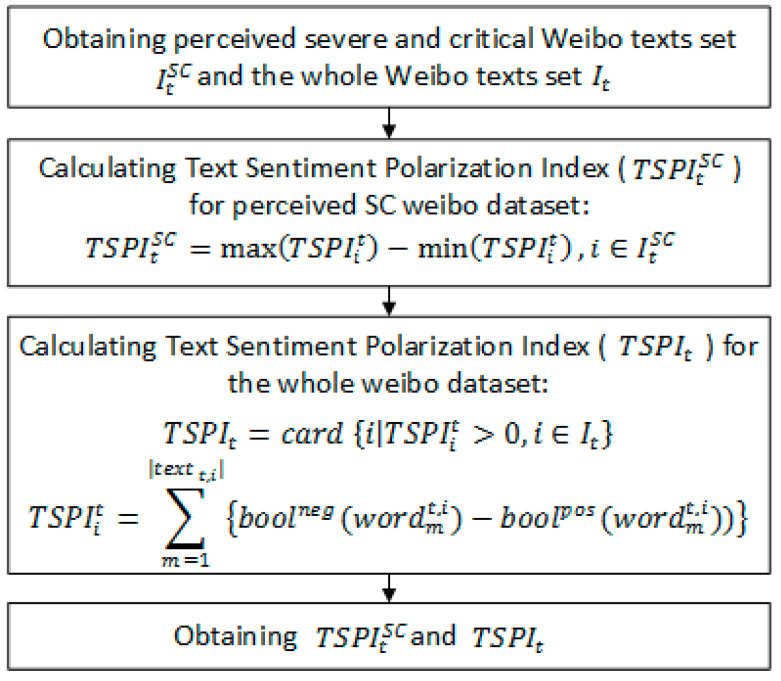
The fourth step of the integrated model.

Note: Figure 2 depicts the four steps of constructing the two crucial prediction indicators of $NPSCP_{t}$. and $TSPI_{t}$. The first step is data preprocessing and the acquisition of seed words. The second step is to extract and expand the feature dictionary of COVID-19 severe and critical patients. The third step is to calculate the number of perceived severe and critical patients and the output $NPSCP_{t}$. The fourth step is to calculate sentiment polarity and the output $TSPI_{t}$ and $TSPI_{t}^{SC}$. The specific process of each step is shown in Figure 3, Figure 4, Figure 5 and Figure 6.

NPSCP is the abbreviation for the number of perceived severe and critical patients in social media data.$\;NPSCP_{t}$ is defined as NPSCP of the t-period. The severe and critical patients are identified based on the constructed feature dictionary. When calculating the number of severe and critical social media texts in the *t*-period, the specific logic is that if there is a word in the social media text that matches any of the words in the feature dictionary, the social media text is recognized as a severe and critical text published by the patient or related parties. The detailed calculation process is as follows:

(1) $$NPSCP_{t}=\sum_{i=1}^{m}\left(bool^{sc}\left({text}_{t,i}\right)\right)$$

(2) $$bool^{sc}\left(text_{t,i}\right)=\{\begin{array}{c} 1,\;\;\;\exists word_{j\;}\;in\;Dic_{sc}\\ \;0,\;\;\;∄\;word_{j\;}\;in\;Dic_{sc} \end{array}$$

where $I_{t}$ is the set of t-period social media texts; $text_{t,i}$ is the set of all words contained in each social media text; $bool^{sc}\left(text_{t,i}\right)$ is a Boolean function, which takes values between {0,1}. If any word in $text_{t,i}$ is included in $Dic_{sc}$, the value takes 1. If there is no word in $text_{t,i}$ included in $Dic_{sc}$, the value takes 0. The detailed calculation process is shown in Figure 2; this model proposes to use social media data to identify potential severe and critical COVID-19 patients; it extracts the feature of the patients through social media sensors and screens out the implied COVID-19 severe and critical patients by user-generated content.

TSPI refers to Text Sentiment Polarity Index, specifically $TSPI_{t}^{SC}$ and $TSPI_{t}$ respectively, representing text sentiment polarity index of social media texts perceived severe and critical related and text sentiment polarity index of all social media texts in the t-period. After data cleaning and recognition of social media texts related to perceived severe and critical patients, we calculated $TSPI_{t}^{SC}$ and $TSPI_{t}$ based on a sentiment dictionary National Taiwan University Semantic Dictionary (NTUSD). NTUSD is a sentiment dictionary based on Simplified Chinese, and it is widely used in sentiment analysis and sentiment modeling in Chinese scenes; it contains 2810 positive words and 8276 negative words. The calculation of $TSPI_{t}^{SC}$ and $TSPI_{t}$ is as follows:

(3) $$TSPI_{t}^{SC}=max\left(TSPI_{i}^{t}\right)-min\left(TSPI_{i}^{t}\right),i\in I_{t}^{SC}\;$$

(4) $$TSPI_{t}=card\;\left\{i|TSPI_{i}^{t}>0,\;i\in I_{t}\right\}$$

(5) $$TSPI_{i}^{t}=\sum_{m=1}^{\left|text_{t,i}\right|}\left\{bool^{neg}\left(word_{m}^{t,i}\right)-bool^{pos}\left(word_{m}^{t,i}\right))\right\}$$

where, $text_{t,i}=\left\{word_{1}^{t,i},word_{2}^{t,i},word_{3}^{t,i},\ldots ,word_{\left|text_{t,i}\right|}^{t,i}\right\}$ represents a certain word list of social media text in t-period after segmentation, $I_{t}$ is for the whole social media data set in the t-period, $I_{t}^{SC}$ is for the severe and critical related social media data set in t-period and function card calculates the cardinal number or size of a set. $bool^{neg}\left(word\right)$ and $bool^{pos}\left(word\right)$, represent the indication function of whether word w is in the NTUSD dictionary. If word w in $NTUSD_{neg}$, then $bool^{neg}\left(word\right)$ equals 1, else 0; it is the same for the value of $bool^{pos}\left(word\right)$. The detailed calculation process is shown in Figure 2; this model proposes the usage of social media text data to measure and profile the negative polarity of Weibo users, especially potential severe and critical COVID-19 patients, to help describe the severity of illness and future trends from a psychological perspective.

$NSCP_{1,t}$ refers to the actual number of severe and critical patients from period 1 to t, $NPSCP_{1,t}$ is the number of perceived severe and critical patients through social media from period 1 to t, and $TSPI_{1,t}$ is the number of negative sentiment texts from the period 1 to t. Let state sequence $L=\left\{l_{1},l_{2},\ldots ,l_{T}\right\}$ indicate the basic state of the number of critical patients in the lockdown area, observation sequence $O=\left\{o_{1},o_{2},\ldots ,o_{T}\right\}$ indicate the number of severe and critical patients that can be observed, and T denote the length of the sequence [26]. The transition between states can be described as a transition matrix, so every element is defined as follows:

(6) $$a_{rv}=P\left(l_{t+1}=q_{v}|l_{t}=q_{r},NSCP_{1,t},NPSCP_{1,t},TSPI_{1,t}\right)$$

where $a_{rv}$ represents the probability transition to state $q_{v}$ at time t+1 under the condition of the state $q_{r}$ at time t, r=1,2,…,N;v=1,2,…,N. Each element in the observation probability matrix can be expressed as $b_{v}\left(o_{g}\right)=P\left(o_{t}|l_{t}=q_{v}\right)$, which represents the probability of generating observation $o_{t}$ under the condition of the state $q_{v}$ at time t. We assume $b_{v}\left(o_{g}\right)$ follows a Gaussian mixture distribution. Let $\pi_{r}=P\left(l_{1}|q_{r}\right)$ is the probability at the state $q_{r}$ when t=1. To capture different amplitude changes, we model the predicted observation sequence after period t+h as:

(7) $$NSCP_{t+h}^{*}=\underset{o_{g}}{argmax}\left[\sum_{r=1}^{N}\sum_{v=1}^{N}\alpha_{g}\left(r\right)a_{rv}b_{v}\left(o_{g}\right)\varphi_{g}\left(v\right)\right],\;\forall g=1,2,\ldots ,h$$

where $\alpha_{g}\left(r\right)=P(o_{1},o_{2},\ldots ,o_{t},r_{t}=q_{r}|a_{rv},b_{v}\left(o_{g}\right),P\left(r_{1}=q_{r}\right))$ is the probability of forward calculation at time t of $q_{r}$, $\varphi_{g}\left(v\right)=P(o_{t+1},o_{t+2},\ldots ,o_{T}|r_{t}=q_{r},a_{rv},b_{v}\left(o_{g}\right),P\left(r_{1}=q_{r}\right))$ is the probability of backward calculation from time t+1 to T of observation sequence. In this work, NSCP, NPSCP*,* and TSPI are included in the transition matrix, which helps further understand the influence of multidimensional observation factors. The range changes of severe and critical patients’ numbers in states can be randomly transferred and captured by the Markov process; this study introduces the Gaussian mixture distribution to calculate an observation matrix that can fully learn the influence of multidimensional observation. For example, the number of severe and critical patients and emotions on Weibo helps to capture the fluctuations in patient numbers.

### 2.3. Ethics Statement

This study was based on an analysis of official data released daily by Hubei Province, China, which are public datasets that are freely available online and social media data from Sina API (Application Programming Interface). We purchased the data collection service from Gooseeker. Gooseeker is an authorized API of Sina Weibo. The data do not involve private information, such as personal name, gender, age, etc., and do not involve privacy and other related issues. All data can be used legally.

## 3. Results

**Figure 7:**
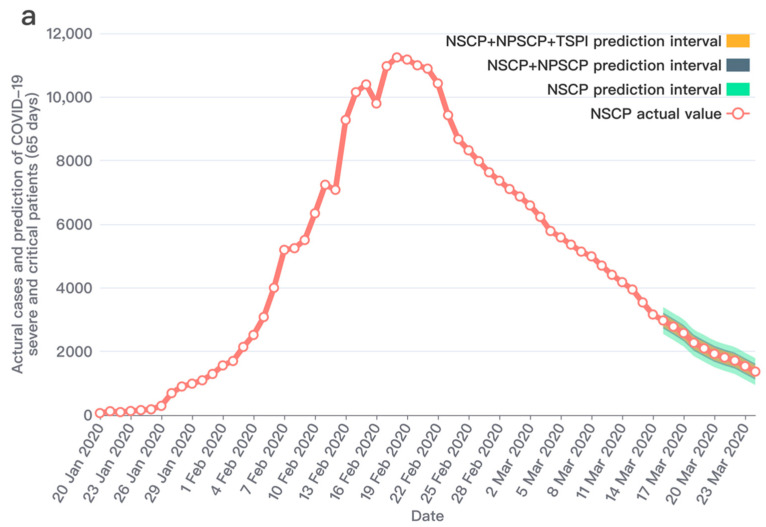

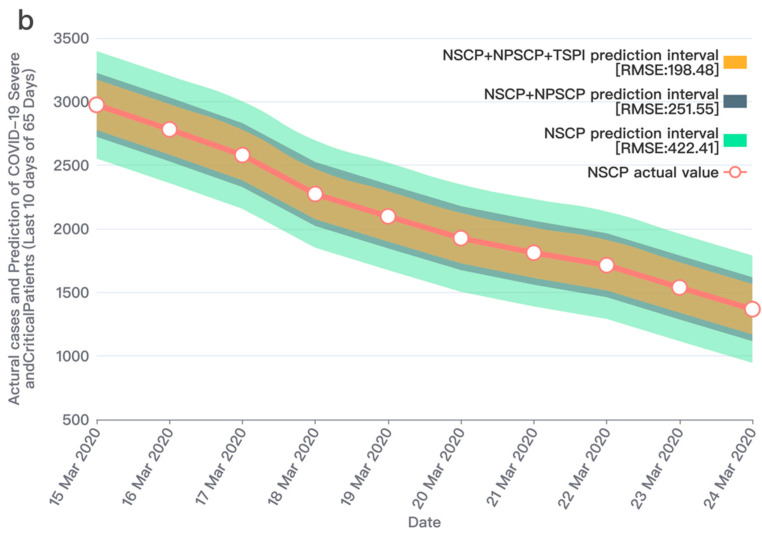
Prediction of the number of severe and critical COVID-19 patients (65 days data set). Note: (**a**) The entire prediction of 65 days during the lockdown period. The first 55 days are the training set and the last 10 days are the test set. The red line with circled points represents the actual values of the number of severe and critical patients; The green area represents the prediction interval obtained from the training data only including $NSCP_{1,55}$, which is based on the red line, RMSE as the fluctuation range. The blue area represents the prediction interval obtained from the training data including $NSCP_{1,55}$ and $NPSCP_{1,55}$, which is based on the red line, RMSE as the fluctuation range. The yellow area represents the prediction interval obtained from the training data including $NSCP_{1,55}$ and $NPSCP_{1,55}$, and $TSPI_{1,55}$, which is based on the red line, RMSE as the fluctuation range. (**b**) The 10 days prediction interval in 65 days. It can be found that the results predicted by the two sets of training data are better than the results of only one set because the RMSE with three sets is 198.48 which is evidently lower than the RMSE 422.41 with one set; this result shows that NPSCP significantly contributes to the prediction. Furthermore, the prediction results (RMSE: 198.48) obtained from the three sets are better than those of the two sets (RMSE: 251.55), which shows that the TSPI has a positive effect in predicting.

**Figure 8:**
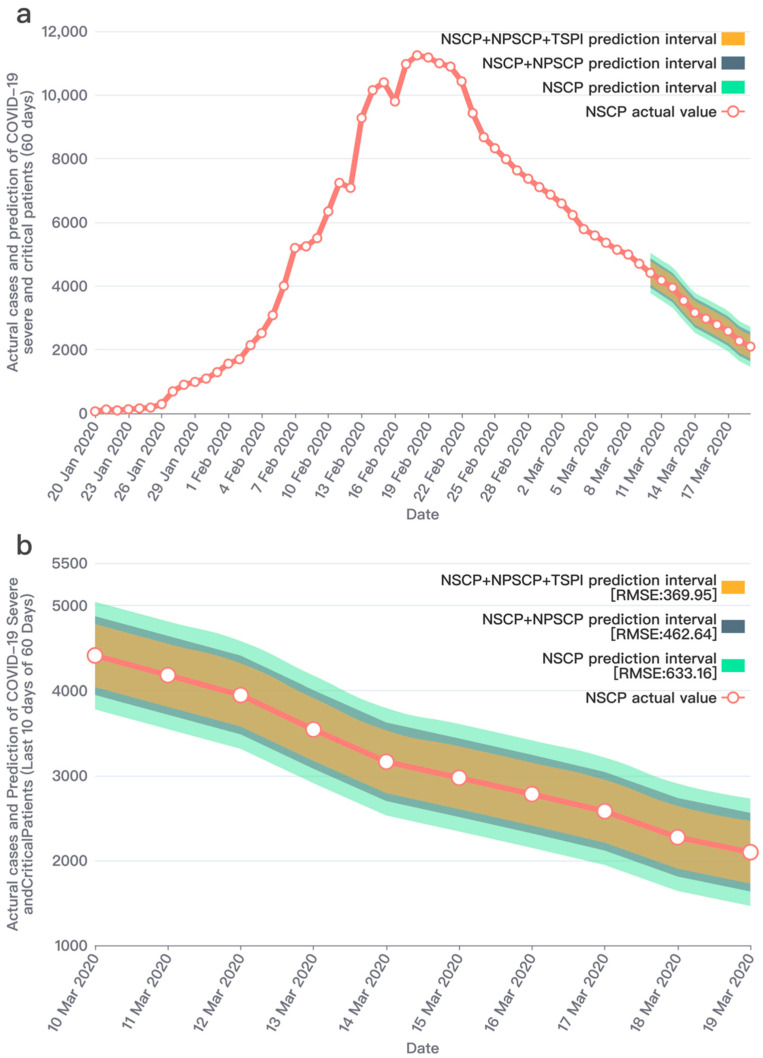
Prediction results of the number of severe and critical COVID-19 patients (60 days data set). Note: (**a**) The entire prediction of 60 days during the lockdown period. The first 50 days are the training set, and the last 10 days are the test set. The legend description is the same as Figure 7a. (**b**) The 10 days prediction interval in 60 days. From the prediction results, we can verify our conclusion that NPSCI and TSPI can help predict the number of severe and critical COVID-19 patients.

**Figure 9:**
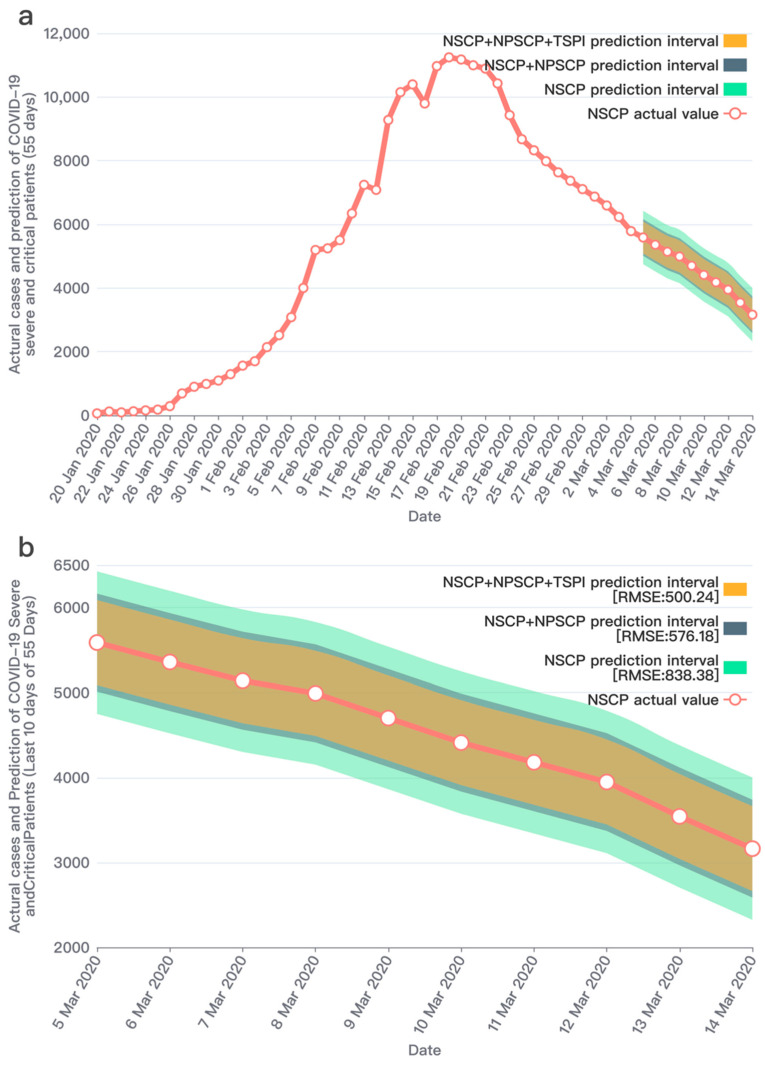
Prediction results of the number of severe and critical COVID-19 patients (55 days data set). Note: (**a**) The entire prediction of 55 days during the lockdown period. The first 45 days are the training set, and the last 10 days are the test set. The legend description is the same as Figure 7a. (**b**) The 10 days prediction interval in 55 days. From the prediction results, we can see that after adding NPSCI and TSPI, our prediction results still improved. Although it is not as good as the 65-day and 60-day performance, we analyze that it is related to limited samples in the training data set and insufficient machine learning.

**Figure 10:**
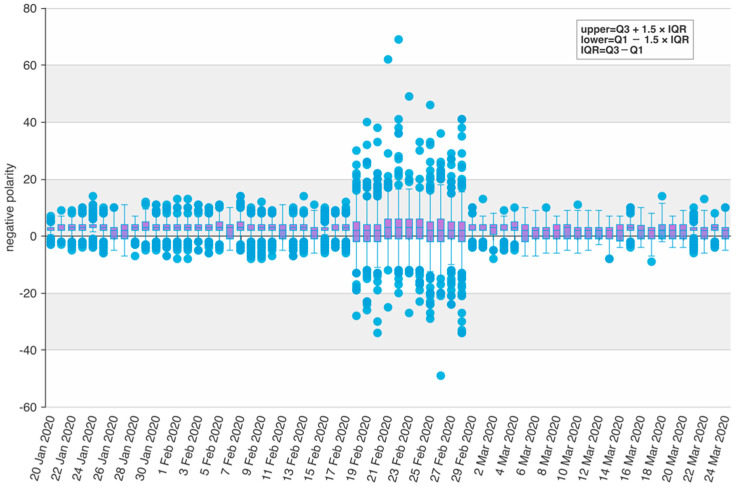
Negative polarity distribution of severe and critical related social media texts in the lockdown period (with outliers). Note: Negative polarity refers to the difference between the number of negative words and positive words in certain social media texts according to the sentiment dictionary NTUSD. During the period from 20 January to 17 February, the overall negative polarity of perceived severe and critical social media texts is negative, and the extreme values are at a relatively stable level with little fluctuation. There is a significant surge from 18 February to 28 February in SC-related negative polarity (the most obvious performance on 22 February), with the span and diversity of emotions increasing significantly, as the upper and lower values of emotions showed. After 28 February, with the gradual control of the epidemic, the overall negative emotions of SC-related microblogs show a gradual reduction trend.

**Figure 11:**
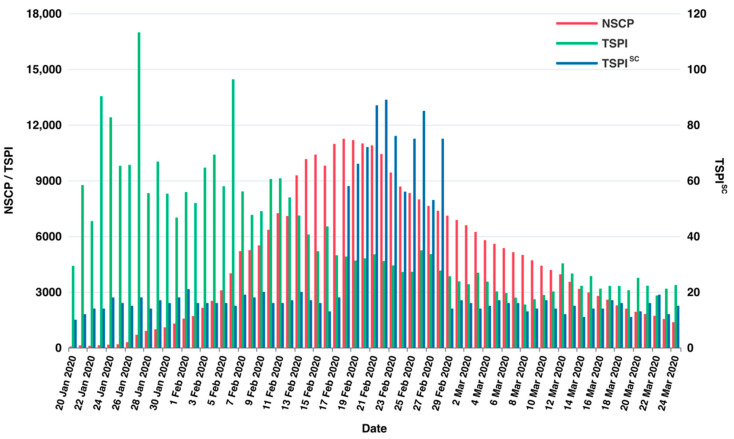
Trends of NSCP, TSPI and $TSPI^{SC}$ during the lockdown period. Note: The trend of TSPI started to rise before the city was locked down (23 January 2020). Both the trend and peak of TSPI are earlier than those of NSCP, indicating that Weibo is an effective social sensor that can reflect the characteristics of COVID-19 patients or potential patients in reality. The peak lag bias of $TSPI^{SC}\;$ may be due to the following reason. Only after the government reported the exact number of COVID-19 patients to the public, did the news media begin to release news about the exact number of COVID-19 patients and the social media discussion also lags behind the release of the news.

Through a significant number of experiments, it showed that the improved prediction model proposed in this study has an outstanding performance after combining the two social media prediction indicators and the officially published data. The prediction performances and results comparison are shown in Figure 7, Figure 8 and Figure 9. We conducted research to calculate the text sentiment polarity index, and present two indexes from two aspects,$\;TSPI_{t}^{SC}$ and $TSPI_{t}$. The two indexes are subject to distinct distributions and have different performances on prediction. The definition of $TSPI_{t}^{SC}$ is based on negative polarity, which indicates the sentiment polarity of each social media text; thus, its trend and distribution fluctuate with $NSCP_{t}$. In the peak period of the epidemic, its fluctuation ranges are large, whilst in other periods, its fluctuation ranges are relatively small. The daily distribution of severe and critical patients related texts’ negative polarity is shown in Figure 10. When using $TSPI_{t}$ as a measure of sentiment polarity, prediction performance is greater than with $TSPI_{t}^{SC}$. Specifically, when using $TSPI_{t}$ as a polarity measure, the RMSE is 198.48, compared with the RMSE of 301.00 when using $TSPI_{t}^{SC}$ with the same parameters. As shown in Figure 11, the trend of $TSPI_{t}$ is not consistent with the trend of $NSCI_{t}$, which is evident from the low Pearson correlation coefficient (−0.31957) between them. Compared with $TSPI_{t}$, the Pearson correlation coefficient between $TSPI_{t}^{SC}$ and $NSCP_{t}$ is higher (0.61314), but $TSPI_{t}^{SC}$ has poorer performance in prediction.

Note: Table 1 shows the prediction results in the forecast horizon. After adding the two indicators of perceived severe and critical patients and the text sentiment polarity index, the RMSEs are gradually decreasing and the predicted values are getting closer to the actual values. Furthermore, this study adjusted the number of training days to conduct comparative experiments and verify the robustness of the model.

This study reveals an interesting phenomenon. When the curves of actual data and perceived social media data are highly consistent, prediction performance is often inadequate. The consistency between the two data series can be described by a Pearson correlation coefficient, and the prediction performance can be manifested by the RMSE indicator. Specifically, a higher Pearson correlation coefficient indicates higher consistency and richness in current information, while a higher RMSE indicates poorer performance in prediction and lack of trend information; therefore, the phenomenon might imply that with abundant current information in perceived social media data and lacking trend information, prediction accuracy may be always adequate. On the contrary, when current information in perceived social media data is lacking but trend information is abundant, it may help to profile future trends and improve prediction performance; thus, this phenomenon suggests that the definition $TSPI_{t}$ contains information on future trends of $NSCP_{t}$, which facilitates the early perception of $NSCP_{t}$.

## 4. Conclusions

By using the social media infodemic to compute the number of perceived severe and critical COVID-19 patients and sentiment polarity, this study enables a more accurate prediction of patients for the horizon period in the lockdown area in comparison to a traditional consensus-based model. Social media data related to severe and critical COVID-19 patients are real-time and publicly available data sources that contain richer information than traditional hospital-reported consensus. Analysis of social media data reveals the reasons why some severe and critical patients did not seek medical treatment in the hospital, including preparing to visit the hospital but not taking action, expecting medical treatment but having no access due to limited hospital beds, having no intention for treatment, or not realizing that they needed treatment. Therefore, the number of perceived severe and critical COVID-19 patients explored through social media contains valuable information that cannot be obtained from traditional hospital consensus statistics. The sentiment polarity of perceived COVID-19 patients from social media includes the emotions concerning themselves, or those around them, or related information in the lockdown area. From the perspective of social sensor theory, social media data depicts the public emotions and opinions concerning COVID-19 patients. The potential evolution and trends in the number of COVID-19 patients were also revealed; this information contains possible trends in severe and critical COVID-19 patients. With a specified horizon perspective, the prediction model proposed in the study can predict the number of perceived severe and critical COVID-19 patients, the text sentiment polarity index, and the number of severe and critical patients in hospitals; this model has several advantages. First, it comprehensively reflects the actual number of severe and critical COVID-19 patients as it includes both the official published data and perceived severe and critical patients by social media. Second, the trend of severe and critical COVID-19 patients receive support from social media public opinion information (COVID-19 sentiment polarity). Further, COVID-19-related social media data can be obtained in real-time at a low cost. To a certain extent, it can complement the shortage of hospital consensus data. Importantly, this model has a specified horizon; this prospect period can increase the preparation time for hospital clinical treatment, medical personnel deployment, and epidemic prevention policy formulation. Theoretically, the prediction model proposed in this paper, which combines actual patient consensus data, social media-perceived patient data, and social media-perceived public sentiment data, is versatile and powerful for application in various research domains including public health and commercial sales; this approach provides a research paradigm based on social media infodemic for building real-time predictions and early warning models in the future.

Practically, the results of this prediction model can play a key role in COVID-19 prevention and control. It can: support hospitals to formulate a clinical treatment plan and optimize the operation of clinical resources; provide support for medical facility manufacturers and reserve companies in arranging production, storage, and transportation; support the Centers for Disease Control and Prevention and other government agencies to formulate epidemic prevention and control policies and strengthen or liberate lockdown measures in epidemic areas; and provide practical guidance for building an epidemic prediction and early warning system based on network sensors.

When people post their emotions and opinions on COVID-19 on the Internet, they also provide rich information to understand the evolution of COVID-19. Data analysis and prediction technology constructed in this paper help to detect the developing trend of prior infectious diseases, and provide data support for further prevention and control. In terms of directions for future work, it is worth trying to develop further experiments to predict outbreaks in multiple lockdown areas and compare the epidemic situation with different characteristics.

## Figures and Tables

**Table 1:** RMSEs with different training days and HMM observed variables.

Number of Days	HMM Observed Variables	RMSE
65	NSCP	422.41
	NSCP+NPSCP	251.55
	NSCP+NPSC+TSPI	198.48
60	NSCP	633.16
	NSCP+NPSCP	462.65
	NSCP+NPSC+TSPI	369.96
55	NSCP	838.38
	NSCP+NPSCP	576.18
	NSCP+NPSC+TSPI	500.25

## Data Availability

The data presented in this study are available on request from the corresponding author.
